# Pectic homogalacturonan masks abundant sets of xyloglucan epitopes in plant cell walls

**DOI:** 10.1186/1471-2229-8-60

**Published:** 2008-05-22

**Authors:** Susan E Marcus, Yves Verhertbruggen, Cécile Hervé, José J Ordaz-Ortiz, Vladimir Farkas, Henriette L Pedersen, William GT Willats, J Paul Knox

**Affiliations:** 1 Centre for Plant Sciences, Faculty of Biological Sciences, University of Leeds, Leeds LS2 9JT, UK; 2 Slovak Academy of Sciences, Institute of Chemistry, Centre of Excellence GLYCOBIOS, Dubravska cesta 9, SK-84538 Bratislava, Slovakia; 3 Department of Biology, University of Copenhagen, Copenhagen Biocentre, Ole Maaløes Vej 5, DK-2200, Copenhagen, Denmark

## Abstract

**Background:**

Molecular probes are required to detect cell wall polymers *in-situ *to aid understanding of their cell biology and several studies have shown that cell wall epitopes have restricted occurrences across sections of plant organs indicating that cell wall structure is highly developmentally regulated. Xyloglucan is the major hemicellulose or cross-linking glycan of the primary cell walls of dicotyledons although little is known of its occurrence or functions in relation to cell development and cell wall microstructure.

**Results:**

Using a neoglycoprotein approach, in which a XXXG heptasaccharide of tamarind seed xyloglucan was coupled to BSA to produce an immunogen, we have generated a rat monoclonal antibody (designated LM15) to the XXXG structural motif of xyloglucans. The specificity of LM15 has been confirmed by the analysis of LM15 binding using glycan microarrays and oligosaccharide hapten inhibition of binding studies. The use of LM15 for the analysis of xyloglucan in the cell walls of tamarind and nasturtium seeds, in which xyloglucan occurs as a storage polysaccharide, indicated that the LM15 xyloglucan epitope occurs throughout the thickened cell walls of the tamarind seed and in the outer regions, adjacent to middle lamellae, of the thickened cell walls of the nasturtium seed. Immunofluorescence analysis of LM15 binding to sections of tobacco and pea stem internodes indicated that the xyloglucan epitope was restricted to a few cell types in these organs. Enzymatic removal of pectic homogalacturonan from equivalent sections resulted in the abundant detection of distinct patterns of the LM15 xyloglucan epitope across these organs and a diversity of occurrences in relation to the cell wall microstructure of a range of cell types.

**Conclusion:**

These observations support ideas that xyloglucan is associated with pectin in plant cell walls. They also indicate that documented patterns of cell wall epitopes in relation to cell development and cell differentiation may need to be re-considered in relation to the potential masking of cell wall epitopes by other cell wall components.

## Background

Cell walls are major components of plant cells that impact significantly on the modes of cell development and the growth and the mechanical properties of plant organs. Plant cell walls are also of considerable economic significance in that they are major components of terrestrial biomass and of plant-derived materials that are used for fibre, fuel and food. Primary and secondary cell walls are comprised of sets of polysaccharides of considerable structural complexity and diversity [1-3]. The major polysaccharide classes are cellulose, hemicelluloses (or cross-linking glycans) and pectic polysaccharides with the latter two classes containing a diversity of polymer structures. In order to understand how specific configurations of polysaccharides and their interactions and associations constitute diverse cell wall structures and functions, methodologies are required to assess polymers *in-situ *throughout organs and within cell walls. Tagged proteins, with the capacity to specifically bind to a structural motif of a polysaccharide, are currently one of the best ways to do this. These proteins are most notably monoclonal antibodies and carbohydrate-binding modules. Cell wall probes, directed to some structural features of polymers of the three major polysaccharide classes have indicated that the occurrence of cell wall polysaccharide structures can be highly regulated in relation to developmental context [4-10]. However, probes are not yet available for all the structural motifs known to occur within cell wall components and thus *in-situ *locations of all polymer structures cannot yet be determined.

Xyloglucans are one of the most abundant hemicelluloses of the primary cell walls of non-graminaceous species and are proposed to have a functional role in hydrogen bonding to and tethering the cellulose microfibrils together. This load-bearing hemicellulosic network maintains the strength of primary cell walls which is a crucial factor underpinning expansive plant growth [1-3,11,12]. The xyloglucan set of hemicelluloses is highly diverse and displays significant taxonomic variation in structure [1,12-15]. Xyloglucans have a backbone of (1→4)-β-D-glucan and some glucosyl residues are substituted with short side chains. A structure-based nomenclature has been developed for xyloglucan-derived oligosaccharides to indicate the attachments to backbone glucosyl sequences [16]. For example, an unbranched glucosyl residue is designated G, a glucosyl residue bearing a single xylose is designated X and one bearing a disaccharide of β-Gal-(1,2)-α-Xyl is designated L. Xyloglucans are classified as XXXG or XXGG type based on the number of backbone residues that carry side chains with the XXXG type having three consecutive glucosyl residues with xylose attached and a fourth unbranched residue [17]. To date it has not been easy to put this structural complexity into cell biological context as only few probes are available. An antiserum to xyloglucan [18] and a monoclonal antibody (CCRCM1) that binds to a fucosylated epitope that is carried by xyloglucan [19] have been developed. These have been used to detect xyloglucan *in-situ *[4,7,20-22].

Here we describe the coupling of a heptasaccharide with 3 xylosyl and 4 glucosyl residues (XXXG in xyloglucan nomenclature) obtained from tamarind seed xyloglucan to a protein carrier to act as an immunogen. Subsequent to immunization we have identified a rat monoclonal antibody, designated LM15, that binds to the XXXG motif of xyloglucan and we have used this antibody to demonstrate the regulation of xyloglucan structure and occurrence within cell walls and in relation to plant anatomy in a range of species.

Polysaccharides do not exist in isolation in plant cell walls and understanding the links and associations between classes of polymers is an important goal to increase our understanding of plant cell wall biology. Biochemical evidence is accumulating that xyloglucan can be attached to pectic polymers in plant cell walls [23-27]. Using enzymatic degradation to remove the pectic homogalacturonan (HG) from cell walls of transverse sections of plant materials in conjunction with the LM15 anti-xyloglucan monoclonal antibody we have demonstrated the existence of developmentally regulated sets of xyloglucan epitopes within cell walls that are masked by the presence of HG. This observation has significant implications for our understanding of the precise developmental patterns of occurrence of xyloglucan in cell walls and of cell wall biology in general.

## Results

### Selection of a XXXG-directed xyloglucan monoclonal antibody

**Figure 1:**
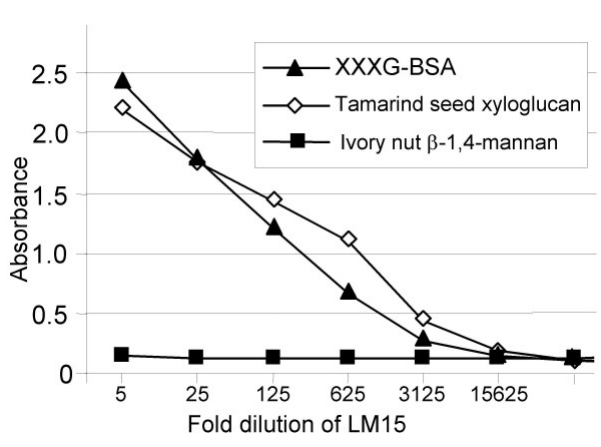
**ELISA of rat monoclonal antibody LM15 binding to the XXXG-BSA glycoprotein used as immunogen, tamarind xyloglucan and mannan**. Absorbance values shown are duplicate means with the standard deviations being ≤ 0.05 absorbance units. The result shown is representative of at least three separate experiments. LM15 bound effectively to the immunogen and tamarind xyloglucan but did not bind to ivory nut mannan. Polysaccharides were coated on to plates by incubation at 50 μg/ml. The LM15 hybridoma supernatant displayed effective recognition of the glycoprotein and tamarind xyloglucan when diluted 500-fold.

**Figure 2:**
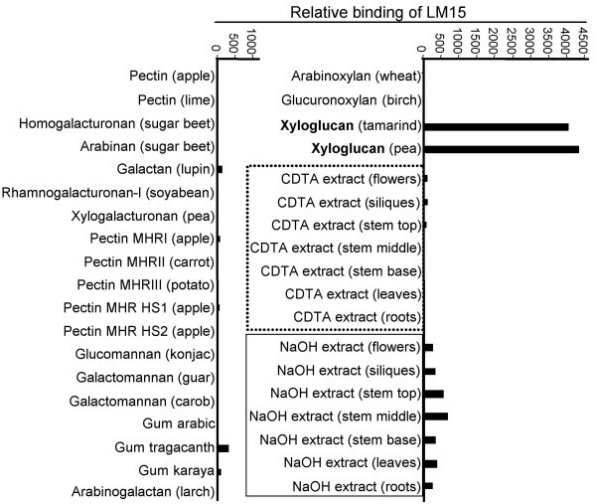
**Microarray analysis of LM15 binding to an array of plant cell wall polysaccharides and CDTA- and NaOH-solubilised isolates of Arabidopsis cell walls**. Pectic and hemicellulosic polysaccharides from a range of species were microarrayed on nitrocellulose sheets and the relative binding of LM15 determined using an enzyme-linked secondary antibody detection system. Binding intensities were quantified and the horizontal scale indicates relative binding. The result shown is representative of at least three separate glycan profiling assays.

To understand xyloglucan structure in the context of plant cell development a set of probes covering aspects of xyloglucan structure is required. As the XXXG oligosaccharide is a major motif of xyloglucans a specific monoclonal antibody probe directed to this motif was generated. The XXXG heptasaccharide from tamarind seed xyloglucan was coupled to BSA by reductive amination. Subsequent to immunization with this neoglycoprotein immunogen, fusion products were screened by ELISAs using tamarind xyloglucan as the antigen. Several cell lines were selected that bound effectively to tamarind xyloglucan and one, secreting a rat IgG2c monoclonal antibody and designated LM15, was selected for full characterization. The binding of LM15 to tamarind seed xyloglucan and the XXXG-BSA neoglycoprotein in an ELISA is shown in Fig. 1. To determine the specificity of LM15 recognition of xyloglucan and to assess its possible binding to other cell wall polymers, LM15 was used to probe glycan microarrays in which large numbers of plant cell wall polysaccharides were assembled [28]. A representative result is shown in Fig. 2 and indicates that there is little recognition of cell wall polymers other than a sample of tamarind xyloglucan (that is non-fucosylated) and pea xyloglucan (that contains fucosylated side chains). The low level of binding to pectic polymer samples and gums tragacanth and karaya is possibly due to low levels of xyloglucan in these extracted samples. Samples of extracts of organs of the model plant *Arabidopsis thaliana *were also included in the microarray [28] and recognition of these was greater for the alkali-solubilised materials rather than cation chelator-solubilised materials – a pattern that would be expected for xyloglucan [28].

**Figure 3:**
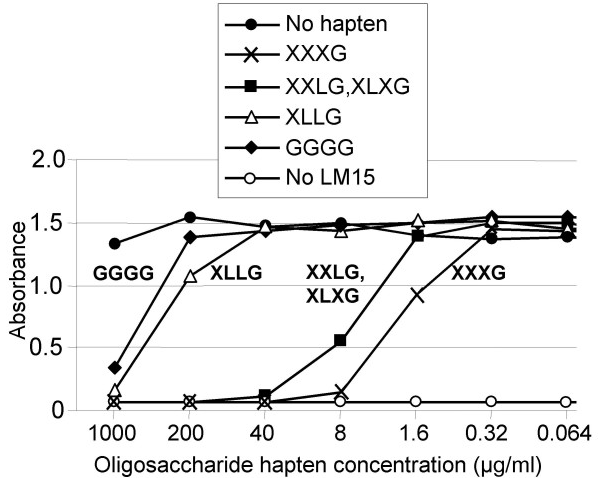
**Competitive-inhibition ELISAs of LM15 binding to tamarind xyloglucan with xyloglucan oligosaccharide haptens**. Absorbance values shown are duplicate means with the standard deviations being ≤ 0.05 absorbance units. The result shown is representative of at least three separate experiments. LM15 was used at a 100-fold dilution and hapten oligosaccharides tested at five-fold dilutions from 1 mg/ml.

The binding of LM15 to tamarind seed xyloglucan in ELISA and in the context of the glycan microarray indicated that it bound specifically to this class of cell wall polymer. In order to confirm this and to determine the structural features required for binding, the inhibition of LM15 binding to tamarind seed xyloglucan by the presence of a range of xyloglucan-derived and related oligosaccharides was determined using competitive-inhibition (hapten) ELISAs. When LM15 hybridoma supernatant was used at 100-fold dilution in an assay with tamarind seed xyloglucan-coated ELISA plates, the most effective hapten inhibitor was the XXXG oligosaccharide as shown in a representative competitive inhibition ELISA in Fig. 3. The concentration of the XXXG oligosaccharide required to inhibit LM15 binding by 50% was 1.9 μg/ml. Equivalent inhibition was achieved with 4.3 μg/ml of a mixture of XXLG and XLXG isomers (that cannot be readily separated), 348 μg/ml XLLG and 560 μg/ml of the GGGG cellotetraose. These data indicate that the presence of two galactosyl residues to a large extent prevented recognition of the xyloglucan oligosaccharides by LM15, although one galactosyl residue was more readily tolerated. Isoprimeverose (α-Xyl(1→6)Glc) and a xylose disaccharide (β-Xyl(1→4)Xyl) had no impact on LM15 binding to xyloglucan when present at 1 mg/ml. In summary, we have generated a monoclonal antibody to the XXXG structural motif of xyloglucan. This probe was used to explore xyloglucan structure and occurrence in relation to plant cell types and cell wall microstructures.

### Xyloglucan in cell walls of cotyledon parenchyma of tamarind and nasturtium seeds

Xyloglucan is known to be a major structural component of the cell walls of the cotyledon parenchyma of tamarind and nasturtium seeds [29-31]. To explore the binding of the XXXG-directed monoclonal antibody LM15 to plant cell walls, excised cotyledon parenchyma tissue from mature seeds of these two species were fixed and embedded in resin and sectioned prior to indirect immunofluorescence analysis. The mouse monoclonal antibody CCRCM1 [19] that binds to a fucosylated epitope of xyloglucan (not present in tamarind seed xyloglucan but present in nasturtium seed xyloglucan) was used for comparison with LM15.

**Figure 4:**
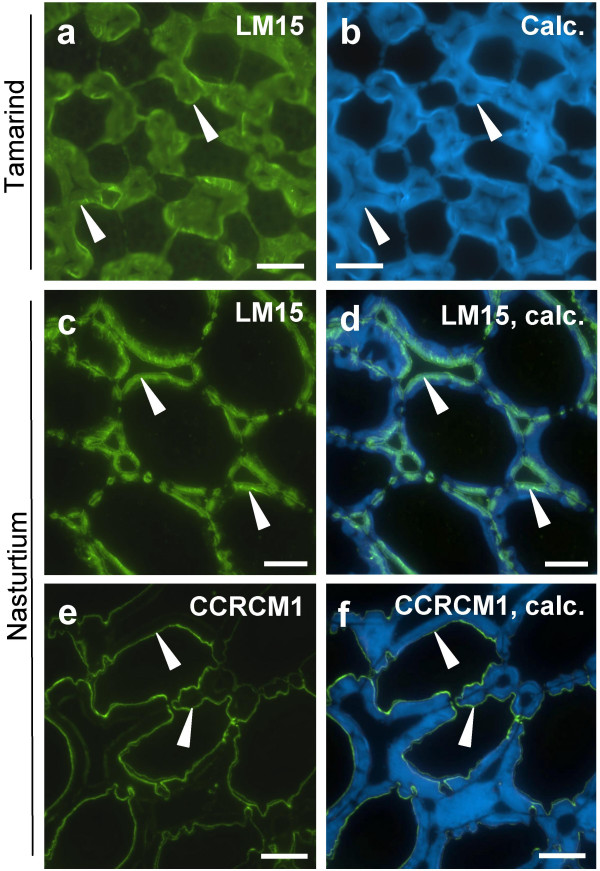
**Indirect immunofluorescence detection of xyloglucan epitopes in sections of tamarind and nasturtium seed cotyledon parenchyma**. a. LM15 binding to cell walls of tamarind cotyledon parenchyma cells. b. Same section stained with Calcofluor White showing extent of cell walls. c. LM15 binding to cell walls of nasturtium cotyledon parenchyma cells. d. Micrograph c combined with Calcofluor White fluorescence. e. CCRCM1 binding to cell walls of nasturtium cotyledon parenchyma cells. f. Micrograph e combined with Calcofluor White fluorescence. Arrowheads in a and b indicate inner cell wall. Arrowheads in c and d indicate inner edge of abundant LM15 immunolabelling. Arrowheads in e and f indicate inner cell wall and region of CCRCM1 immunolabelling. Scale = 20 μm.

A comparison of LM15 binding and Calcofluor White staining of tamarind seed cotyledon parenchyma indicated that LM15 bound throughout the cell walls, including the extensive cell wall thickenings of the cotyledon parenchyma (Fig. 4). In contrast, CCRCM1 did not bind to these cell walls although it did bind to non-thickened cell walls of the vascular tissue (not shown). Tamarind belongs to the family Fabaceae in the order Fabales. Xyloglucan is also an abundant storage polymer in seeds of nasturtium belonging to the family Tropaeolaceae in the order Brassicales. The binding of LM15 and CCRCM1 to nasturtium seed cotyledon parenchyma cell walls is shown in Fig. 4 and indicates recognition of distinct regions of the seed parenchyma cell walls. LM15 bound to the thickened regions of cell walls and most strongly to the outer regions next to middle lamellae (Fig. 4c,d). In contrast, the CCRCM1 epitope was less abundant and variable in occurrence and when present it was exclusively in a thin region of the inner cell walls facing the plasma membrane (Fig. 4e,f).

### LM15 binding to cell walls of tobacco and pea stems is increased by a pre-treatment with pectate lyase to remove pectic homogalacturonan

**Figure 5:**
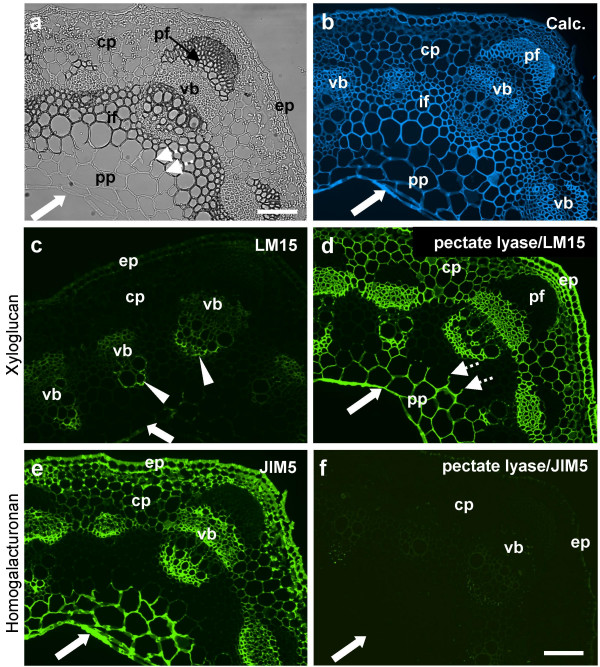
**Indirect immunofluorescence detection of xyloglucan and pectic HG epitopes in TS pea stem internode**. a. Bright field image showing anatomy. b. Calcofluor White image of section shown in a showing all cell walls. c. LM15 binding to an equivalent section. The antibody binds most strongly to distal region of protoxylem (arrowheads). d. An equivalent section to c that had been pre-treated with pectate lyase to remove pectic HG. LM15 binds strongly to epidermal and parenchyma cell walls. e. Section immunolabelled with pectic HG probe JIM5. f. Equivalent section to e pre-treated with pectate lyase indicates that the JIM5 epitope had been abolished. Arrows indicate inner edge of pith parenchyma. Dotted shaft arrows indicate junction between non-sclerifed parenchyma cell walls with sclerified parenchyma/fibre cell walls. cp = cortical parenchyma, pp = pith parenchyma, ep = epidermis, vb = vascular bundle, pf = phloem fibre bundle. Scale = 100 μm.

The structure of xyloglucans is taxonomically diverse with species such as pea and arabidopsis, belonging to the Rosidae, having a XXXG type xyloglucan [1,3]. Bright field and Calcofluor White stained images of a region of a transverse section through a pea stem internode show a portion of the ring of vascular bundles (with larger bundles at stem corners) linked through interfascicular regions by a band of sclerified parenchyma or fibre cells and cortical and pith parenchymas in distal and proximal regions respectively (Fig. 5a,b). Indirect immunofluorescence analysis of LM15 binding to an equivalent section of pea stem indicated that this antibody bound to a limited set of cell walls in the inner protoxylem regions of vascular bundles (Fig. 5c). There was also some weak binding to cells in the region of the phloem of the vascular bundles and to the pith parenchyma. Omitting LM15 from the immunolabelling procedure on an equivalent section resulted in no observed fluorescence (not shown). This unexpectedly low level of recognition of the cell walls by LM15 was explored further by removal of pectic HG from sections by a pre-treatment with a recombinant pectate lyase. This pre-treatment led to a large increase in the detection of the LM15 epitope across the section and in a distinct pattern of cell walls as shown in Fig. 5d, i.e. the pre-treatment of the section had exposed a set of LM15 epitopes. The pectate lyase pre-treatment resulted in the LM15 epitope being detected in abundance in the epidermal cell walls, those of the cortical and pith parenchymas and cells in the phloem region of the vascular bundles and the inner regions of the xylem strands. In pith parenchyma cells directly adjacent to cells with thickened cell walls in the interfascicular regions the occurrence of the LM15 epitope stopped abruptly at the junction with the fibre cell (Fig. 5d) indicating that only three of the four walls of a cell in section were labelled by the antibody. In summary, the LM15 epitope was detected in most cells other than cells with secondary cell walls (sclerenchyma fibres of the vascular bundles and the interfascicular fibres). Similar patterns of occurrence, before and after enzyme treatment were observed for the CCRCM1 epitope (not shown). This pattern of occurrence after enzyme treatment is similar to that of pectic HG in an equivalent section as shown by the binding of anti-HG monoclonal antibody JIM5 [32] to a section not treated with the enzyme in Figure 4e. All control sections in this study not treated with pectate lyase were incubated with the high pH buffer required for maximal enzyme action. The high pH results in de-esterification of pectic HG and therefore, in this case, JIM5 indicates the presence of HG present in cell walls independent of original methyl-esterification status. The loss of the JIM5 HG epitope from the section as a consequence of pectate lyase treatment is shown for comparison in Fig. 5f.

**Figure 6:**
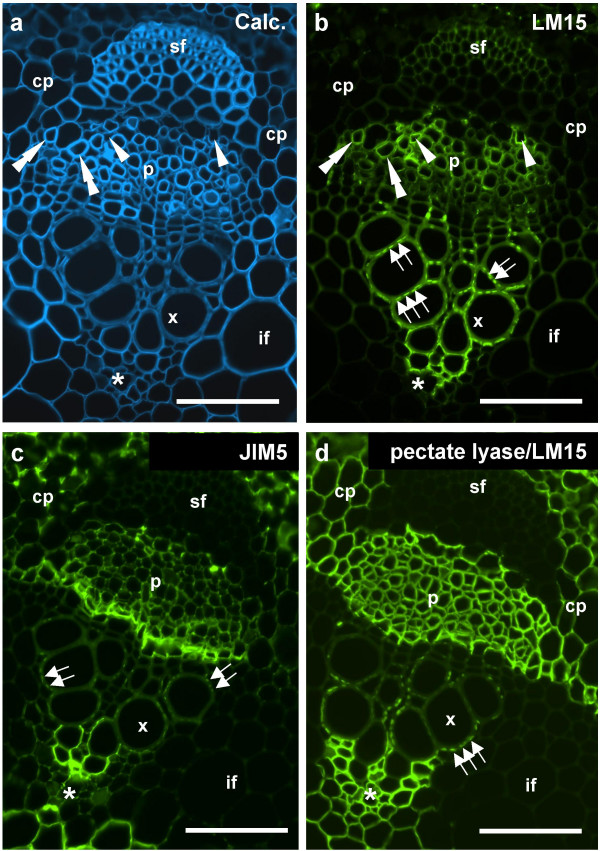
**Indirect immunofluorescence detection of xyloglucan and pectic HG epitopes in TS pea stem internode vascular bundles**. a. Calcofluor White image of section showing all cell walls. b. LM15 binding to an equivalent section to a. The antibody binds most strongly to a region of protoxylem but also certain cells in the phloem region. c. JIM5 binding to an equivalent section shows binding to protoxylem and cambial cells. d. LM15 binding to an equivalent section pre-treated with pectate lyase shows the epitope detected abundantly in the phloem/cambial regions and cortical parenchyma. Arrowheads indicate cells in the phloem regions without thickened cell walls in which the LM15 epitope is detected without pre-treatment. Double arrowheads indicate cells with thickened cell walls/LM15 epitope in the phloem region. Sets of arrows indicate the punctuate presence of LM15 and JIM5 epitopes in xylem vessel cell walls. Asterisk indicates distal extent of the protoxylem. cp = cortical parenchyma, p = phloem region, pf = phloem fibre bundle, x = xylem vessel, if = interfascicular fibres. Scale = 10 μm.

At a tissue level the LM15 epitope unmasked by pectate lyase action and the JIM5 epitope on an untreated section have a similar occurrence. To document the occurrence of the LM15, pectate-lyase-unmasked LM15 and JIM5 epitopes in more detail higher magnification micrographs of vascular bundles are shown in Fig. 6. In an untreated section the LM15 epitope was most abundant in cell walls of protoxylem cells, discrete domains of metaxylem cell walls and also certain cells of the phloem region that can also be identified from Calcofluor White labelling to have in some cases thickened cell walls and in other cases not (Fig. 6b). In addition, the epitope was detected in cells, with the characteristics of parenchyma cells, adjacent to the smallest protoxylem cells. After pectate lyase treatment the occurrence of labelling was maintained along with the strong labelling of all cell walls in the phloem region and the cortical and pith parenchymas. The pattern of the JIM5 epitope was similar in that it was also present in discrete domains of metaxylem cell walls but showed subtle distinctions in that it was abundant in the cambial layer between the xylem and phloem regions. In the phloem region the JIM5 epitope did not appear throughout cell walls but at intercellular regions and was also relatively more abundant at cell corners in the cortical parenchyma in comparison with the LM15 epitope.

**Figure 7:**
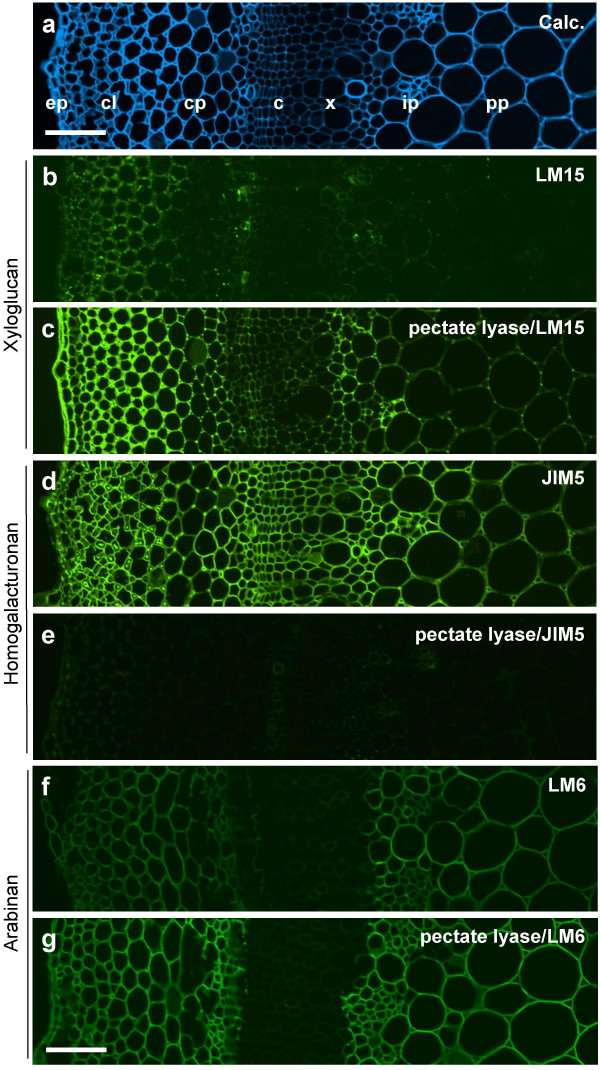
**Indirect immunofluorescence detection of xyloglucan, pectic HG and arabinan epitopes in TS tobacco stem internode**. a. Calcofluor White image of section showing cell types from epidermis to pith parenchyma. b. LM15 binding to an equivalent section to a. There is weak recognition of cortical collenchyma/parenchyma and isolated groups of cells in the cambial region. c. An equivalent section to b that had been pre-treated with pectate lyase to remove pectic HG. LM15 binds strongly to epidermal and parenchyma cell walls. d. Section immunolabelled with pectic HG probe JIM5 which binds to all cell walls. e. Equivalent section to d pre-treated with pectate lyase indicates that the JIM5 epitope has been abolished. f. Section immunolabelled with arabinan probe LM6 which binds most strongly to cell walls of cortical and pith parenchyma. g. Equivalent section to f pre-treated with pectate lyase indicates increased detection of the same pattern of the LM6 epitope. ep = epidermis, cl = collenchyma, cp = cortical parenchyma, c = cambium, x = xylem, ip = internal phloem, pp = pith parenchyma. Scale = 100 μm.

Solanaceous species, belonging to the Solanales of the Asteridae, do not have XXXG xyloglucan but have distinct xyloglucan oligosaccharides of type XXGG and XSGG where S indicates an arabinosyl residue attached to the xylosyl residue [1,13,33]. LM15 bound weakly to cell walls in sections of tobacco stem as shown in Fig. 7 and this was mostly to collenchyma, distinct periodic cells in the cambium and pith parenchyma adjacent to the band of xylem (Fig. 7b). Pre-treatment of an equivalent section with pectate lyase to remove pectic HG increased LM15 binding. After HG removal, the LM15 epitope was detected throughout cell walls of the epidermis, the cortical collenchyma and parenchyma and also the cambium and pith parenchyma (Fig. 7c). Monoclonal antibody CCRCM1 did not bind to tobacco stems before or after pectate lyase treatment (not shown). Labelling of sections with anti-HG JIM5 before and after section treatment with pectate lyase indicated that HG epitopes were effectively removed from the section (Fig. 7d,e) and also indicated that the JIM5 HG epitope occurred more widely than the LM15 epitope in all cell walls of all cell types throughout the organ. To explore how a pectate lyase pre-treatment impacts on the capacity to detect other cell wall epitopes in this system, equivalent untreated and treated sections were probed with monoclonal antibody LM6 directed to the (1→5)-α-L-arabinan, a structural motif that is often associated with the rhamnogalacturonan-I polysaccharide of pectin [34]. This antibody bound in a similar pattern to epidermal cells, cortical and pith parenchymas before and after treatment with pectate lyase although the fluorescence signal was more intense after pectate lyase treatment (Fig. 7f,g).

**Figure 8:**
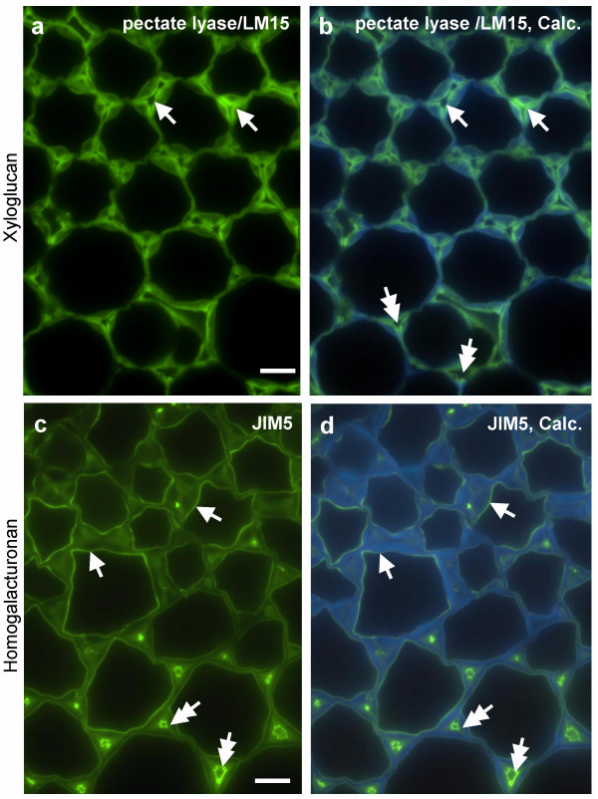
**Indirect immunofluorescence detection of xyloglucan and pectic HG epitopes in TS tobacco stem internode showing higher magnification micrographs of region of cortical collenchyma/parenchyma**. a. LM15 binding to cell walls after pectate lyase pretreatment is most strong at outer regions adjacent to middle lamellae. b. Same section as a shown combined with Calcofluor White fluorescence. c. Equivalent section to a immunolabelled with JIM5 showing HG across cell walls and abundance at inner cell walls and intercellular spaces of parenchyma (bottom of micrograph). d. Same section as c shown combined with Calcofluor White fluorescence. Arrows in a and b indicate outer cell wall regions adjacent to middle lamellae with abundant LM15 epitope. Arrows in c and d indicate inner edge of cell walls and abundant JIM5 epitope. Double headed arrows indicate intercellular spaces. Scale = 10 μm.

**Figure 9:**
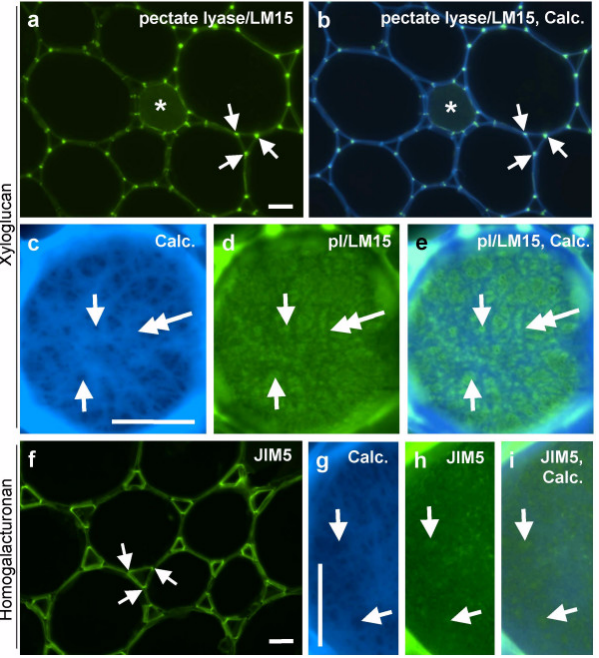
**Indirect immunofluorescence detection of xyloglucan and pectic HG epitopes in TS tobacco stem internode showing higher magnification micrographs of region of pith parenchyma**. a. LM15 binding to cell walls after pectate lyase pretreatment is most strong at points of cell adhesion at corners of an intercellular space (arrows). b. Same section as a shown combined with Calcofluor White fluorescence. c. Calcofluor White fluorescence of an intact cross wall in pith parenchyma – equivalent cell shown as an asterisk in a and b. d. Same cell as c showing LM15 binding. e. Combined image of c and d. Single-headed arrows in c, d and e indicate points of weaker Calcofluor White/stronger LM15 fluorescence. Double-headed arrows in c, d and e indicate points of stronger Calcofluor White/weaker LM15 fluorescence. f. Equivalent section immunolabelled with JIM5 showing epitope particularly abundant around the entire lining of intercellular spaces. Arrows indicate points of cell adhesion at corners of an intercellular space. g. Equivalent section to c. h. Same section as g showing JIM5 labelling. i. Combined image of g and h. Arrows in g, h and i indicate cell wall regions with weaker Calcofluor fluorescence. Scale = 10 μm.

A more detailed comparative analysis of the LM15 and JIM5 epitopes in cortical regions where collenchyma cells merge into parenchyma cells is shown in Fig. 8. The LM15 xyloglucan epitope, revealed by pectate lyase pre-treatment, occurred throughout collenchyma wall thickenings but was most abundant in cell wall regions adjacent to middle lamellae, a pattern of occurrence that was maintained in the cortical parenchyma (Fig. 8). In contrast, the JIM5 HG epitope was distributed evenly across thickened collenchyma cell walls and some cases a stronger abundance at the inner cell wall adjacent to the plasma membrane was observed. The epitope was particularly abundant in cell walls lining the intercellular spaces in the parenchyma cells. In the pith parenchyma (Fig. 9), enzymatic removal of pectic HG uncovered the LM15 epitope throughout the cell wall but a particularly abundant occurrence was always observed in this tissue at the junctions between adhered and unadhered cell walls at intercellular spaces (Fig. 9a). In contrast, the JIM5 HG epitope was most abundant in cell walls lining the entire intercellular spaces (Fig. 9f). In the section preparation used for this study, certain cells were viewed not only in section across longitudinal walls but were observed in addition to present intact inner surfaces of transversal cell walls – see asterisk in Fig. 9a. Immunocytochemical analysis of such walls can provide an additional insight in to cell wall microstructures [35]. Higher magnification of these regions of the inner face of cell walls with Calcofluor White fluorescence (Figs. 9c-e) revealed regions with reduced fluorescence, that are possibly pit fields. These regions displayed more LM15 fluorescence relative to regions showing strong Calcofluor White fluorescence (Figs 9c–e). A similar differential labelling of regions showing strong and weak fluorescence with Calcofluor White was shown by JIM5 although this was less marked (Figs. 9g–i).

## Discussion

We show that immunization with a neoglycoprotein has been successful in the generation of a rat monoclonal antibody, LM15, directed to the XXXG motif of xyloglucan that can bind effectively to tamarind xyloglucan (a polymer without fucosylation) and also to cell walls in a range of species. LM15 can bind to pea cell walls (with XXXG xyloglucan) and also to tobacco stem cell walls with XXGG xyloglucan indicating that the LM15 antibody may not require the entire heptasaccharide XXXG for optimal recognition – a facet of LM15 binding to xyloglucan that was also indicated by hapten oligosaccharide inhibition studies in which a mixture of XXLG and XLXG oligosaccharides was observed to effectively inhibit binding.

Polysaccharide-directed probes such as monoclonal antibodies are important tools to gain an understanding of polysaccharide diversity and occurrence in cell walls which is an essential aspect of understanding the molecular basis of cell wall functions. A xyloglucan antiserum [18] and a monoclonal antibody CCRCM1 [19] have been used to demonstrate developmentally regulated patterns of xyloglucan occurrence in a range of systems [7,21,22]. In sections of nasturtium seed monoclonal antibodies LM15 and CCRCM1 bound to distinct regions of cotyledon parenchyma cell walls indicating the spatial regulation of xyloglucan structure within these cell walls. A region of the nasturtium cell walls between the regions bound by these antibodies (revealed by Calcofluor White fluorescence) was not strongly bound by either probe (and pre-treatment with pectate lyase had no impact on epitope occurrence). To know whether xyloglucan is reduced in amount or the xyloglucan is structurally distinct in these regions requires the development of probes for other structural features of xyloglucans.

Binding of LM15 to untreated transverse sections of stem internodes of pea and tobacco indicated recognition of only a few cell types and often weakly. Binding was shown to be increased considerably by the enzymatic removal of pectic HG. In cell wall immunochemistry studies, it is often assumed that a section through an organ/cell and thus across cell wall layers from the plasma membrane to the middle lamella would expose all polymers present in the cell walls. The implication, from many documented occurrences of cell wall epitopes, has been that a restricted occurrence of an epitope reflected the presence or absence of a particular epitope and this has in some cases been supported by physicochemical analysis of isolated polymers. This is the first report of a clear case of substantial epitope masking where most of the copies of an epitope present in a section have been masked by the presence of another cell wall polymer, in this case pectic HG. It is of considerable interest that, in both pea and tobacco stem internodes, some xyloglucan epitopes were detected without enzymatic removal of pectic HG in addition to those revealed by pectic HG removal. This suggests the possibility of two distinct presentations of the LM15 epitope in relation to pectin in cell walls. These observations have important implications for our understanding of cell wall polymer configurations and should be born in mind when considering the developmental regulation of cell wall structures, as evidenced by antibody probes.

The basis of the uncovering of xyloglucan epitopes by pectic HG removal could be a general increase in cell wall porosity allowing increased access to epitopes or alternatively an intimate specific structural association between pectin and xyloglucan *in muro *that occludes xyloglucan structures. The comparative assessment of the LM6 arabinan epitope in tobacco stem indicated that its detection increased after pectate lyase treatment but not to the extent observed for the LM15 xyloglucan epitope. Moreover, no difference in the pattern of occurrence of the LM6 epitope was revealed. This observation suggests that the pectate lyase pre-treatment can increase cell wall porosity and thus all antibody access to some extent. However, the clear impact on the extent and pattern of LM15 xyloglucan epitope detection indicates a more intimate association between xyloglucan and pectin in this system.

Pectin and xyloglucan are both quantitatively important polymers of plant cell walls comprising approximately a third each of the polysaccharides of primary cell walls of dicotyledons [1]. In terms of cell wall architectures, xyloglucan is known to attach to cellulose microfibrils by means of hydrogen bonds and is proposed to tether adjacent microfibrils providing the mechanical basis of the resisting cell enlargement. The complex pectic network of several polymers (the major one being HG) embeds the cellulose-xyloglucan network and imposes cell wall properties including cell wall porosity [2,36,37]. An early model of cell wall structure proposed a glycosidic link between xyloglucan and a neutral side chain of pectin [38]. Recent evidence has confirmed a link between xyloglucan and acidic pectic polymers but specifically through the rhamnogalacturonan-I domains of the pectic molecules [23-27]. These studies indicate that such links are widespread and, in the case of cultured cells of arabidopsis, evidence has been reported that up to 50% of xyloglucan is synthesized on a pectic primer prior to cell wall deposition and that the interpolymer bonds are stable in the cell wall [26,27]. The significance of glycosidic links between pectic polymers and xyloglucan and the observations reported here are not yet clear.

In cell walls of pea and tobacco stem internodes the pectic HG and the LM15 xyloglucan epitope do not co-localize precisely. It is of interest that the uncovered LM15 xyloglucan epitopes occurred in diverse complex patterns in relation to cell wall features and microstructures in a range of cell types such as parenchyma, metaxylem and collenchyma cells. In many cases the LM15 epitope was not evenly distributed throughout cell walls. At the inner face of transverse walls in the pith parenchyma of tobacco stem the LM15 epitope did not co-localize with Calcofluor White fluorescence (likely to be indicative of cellulose). Other approaches have indicated that cellulose microfibrils may not be evenly coated with xyloglucan, that xyloglucan can occur in distinct cell wall domains and may be minimal in some cell walls [39,40]. It has also been demonstrated that xyloglucan structure changes during cell growth in pea [41] and it is possible that structural changes are spatially regulated within cell walls during cell development. The detection of abundant LM15 xyloglucan epitope at corners of intercellular spaces, shown most strikingly in this study for tobacco pith parenchyma cell walls, is a pattern of occurrence that has been observed for the LM7 pectic HG epitope in a wide range of parenchyma systems [6]. This may indicate, for this tissue, a role for xyloglucan in intercellular space formation or stabilisation, possibly through an association with pectin.

## Conclusion

A novel xyloglucan binding rat monoclonal antibody LM15 has been developed for xyloglucan analysis *in planta*. The demonstration that, in certain organs, large sets of xyloglucan epitopes are masked by the presence of pectic HG has implications for understanding xyloglucan function in primary cell walls and cell wall biology in general. The use of enzymic degradation in conjunction with cell wall probes is likely to be an important analytical tool for the study of the developmental regulation of links between pectic polymers and xyloglucan. Further work will be required to dissect the extent of cell wall epitope masking occurring between other sets of cell wall polysaccharides.

## Methods

### Preparation of neoglycoprotein immunogen, immunization protocol and isolation of a xyloglucan-directed monoclonal antibody

A neoglycoprotein (XXXG-BSA) was prepared by coupling a heptasaccharide containing 3 xylosyl and 4 glucosyl residues (XXXG, Megazyme, Bray, Ireland) to BSA by reductive amination [42]. XXXG (30 mg) was dissolved in 1.0 ml of 0.2 M sodium borate buffer pH 9.0. This was followed by the addition of 20 mg BSA and then 30 mg of sodium cyanoborohydride. The mixture was maintained in a water bath at 50°C with occasional mixing. After 24 h the pH was adjusted to pH 4.0 by the addition of 45 μl of 80% (v/v) acetic acid. The solution was then dialysed extensively against distilled water with several changes over 4 days.

Rat immunization, hybridoma preparation and cloning procedures were performed as described previously [34]. Two male Wistar rats were injected with 100 μg XXXG-BSA in complete Freund's adjuvant administered subcutaneously on day 0, with the same amount administered with incomplete Freund's adjuvant on days 33 and 71. On day 145, a selected rat was given a prefusion boost of 100 μg XXXG-BSA in 1 ml PBS by intraperitoneal injection. The spleen was isolated three days later for isolation of lymphocytes and fusion with rat myeloma cell line IR983F [43]. Antibodies were selected by ELISA using tamarind xyloglucan as antigen. Subsequent characterization was by means of a glycan microarray of cell wall polymers [28] and competitive inhibition ELISAs using the xyloglucan XXXG heptasaccharide from tamarind xyloglucan and a series of related xyloglucan oligosaccharides. A mixture of the XXLG and XLXG octasaccharide isomers and the XLLG nonasaccharide were derived from tamarind xyloglucan as described [44] and purified by HPLC using Tosoh TSK Gel Amide column (21.5 × 300 mm) eluted with 65% aqueous acetonitrile. Cellotetraose GGGG was prepared by acetolysis of cellulose [45] and separated from the mixture of deacetylated oligosaccharides by HPLC as above. The sample of pea xyloglucan was a gift from Marie-Christine Ralet (INRA, Nantes, France). ELISAs were carried out as described previously [6] and in all cases immobilised antigens were coated at 50 μg/ml. Mannan, tamarind xyloglucan polymers, isoprimeverose and xylose disaccharide were obtained from Megazyme, Bray, Ireland. The selected antibody, an IgG2c, was designated LM15.

### Plant materials and immunocytochemistry procedures

Tamarind (*Tamarindus indica *L.) seeds were obtained from Jungle Seeds, Watlington, UK) and nasturtium (*Tropaeolum majus *L. cv Tom Thumb) seeds from Mr. Fothergill's Seeds Ltd., Newmarket, UK. Tamarind and nasturtium seeds were imbibed for 24 h and then pieces of cotyledon parenchyma were excised, fixed and prepared for embedding in LR White resin with subsequent sectioning for indirect immunofluorescence analysis as described previously [8]. Tobacco (*Nicotiana tabacum *L.) and pea (*Pisum sativum *L.) plants were grown in a greenhouse with 16 h days and maintained between 19 and 23°C. Regions of second internodes from the top of six-week old plants were fixed, embedded in wax and sectioned as described previously [46].

In addition to LM15, three further monoclonal antibodies were used in this study using indirect immunofluorescence: CCRCM1, a mouse monoclonal antibody to a fucosylated epitope of xyloglucan [19], a gift from Dr. Michael Hahn (CCRC, University of Georgia, USA), JIM5, a rat monoclonal antibody to methyl-esterified and unesterified epitopes of HG [32] and LM6, a rat monoclonal antibody to arabinan [34]. Section pre-treatment to remove HG from cell walls involved incubation of sections with a recombinant microbial pectate lyase 10A [47] (a gift from Prof. Harry Gilbert, University of Newcastle-upon-Tyne) at 10 μg/mL for 2 h at room temperature in 50 mM *N*-cyclohexyl-3-aminopropane sulfonic acid (CAPS), 2 mM CaCl_2 _buffer at pH 10 as described [10]. The high pH of the enzyme buffer removes HG methyl esters in cell walls and results in HG being susceptible to pectate lyase degradation and also suitable for recognition by JIM5. Sections not treated with the pectate lyase were incubated for an equivalent time with the high pH buffer without enzyme and imaged as untreated controls. After enzyme or buffer treatment, sections were incubated in phosphate-buffered saline (PBS) containing 5% (w/v) milk protein (MP/PBS) and a 5-fold dilution of antibody hybridoma supernatant for 1.5 h. Samples were then washed in PBS at least 3 times and incubated with a 100-fold dilution of anti-rat IgG (whole molecule), or anti-mouse IgG, linked to fluorescein isothiocyanate (FITC, Sigma, UK) in MP/PBS for 1.5 h in darkness. The samples were washed in PBS at least 3 times and incubated with Calcofluor White (0.2 μg/mL) (Fluorescent Brightner 28, Sigma, UK) for 5 min in darkness. Samples were washed at least 3 times and then mounted in a glycerol-based anti-fade solution (Citifluor AF1, Agar Scientific, UK). Immunofluorescence was observed with a microscope equipped with epifluorescence irradiation and DIC optics (Olympus BX-61). Images were captured with a Hamamatsu ORCA285 camera and Improvision Volocity software.

## Authors' contributions

SEM carried out preparation of the immunogen, the cell culture for isolation and cloning of LM15 and its immunochemical characterization. SEM, YV, CH, JJO–O and JPK participated in preparation of plant materials, characterization of antibody binding to sections and acquisition of micrographs. VF carried out the isolation of oligosaccharides. HLP and WGTW carried out microarray analyses. JPK conceived of the study and drafted the manuscript. All authors read and approved the final manuscript.
